# Construction of a disease‐specific lncRNA‐miRNA‐mRNA regulatory network reveals potential regulatory axes and prognostic biomarkers for hepatocellular carcinoma

**DOI:** 10.1002/cam4.3526

**Published:** 2020-11-24

**Authors:** Qi Zhang, Lin Sun, Qiuju Zhang, Wei Zhang, Wei Tian, Meina Liu, Yupeng Wang

**Affiliations:** ^1^ Department of Biostatistics Harbin Medical University Harbin Heilongjiang China

**Keywords:** CeRNA network, differential network analysis, hepatocellular carcinoma, nomogram, prognostic signature

## Abstract

Hepatocellular carcinoma (HCC) is a heterogeneous malignancy with a high incidence and poor prognosis. Exploration of the underlying mechanisms and effective prognostic indicators is conducive to clinical management and optimization of treatment. The RNA‐seq and clinical phenotype data of HCC were retrieved from The Cancer Genome Atlas (TCGA), and differential expression analysis was performed. Then, a differential lncRNA‐miRNA‐mRNA regulatory network was constructed, and the key genes were further identified and validated. By integrating this network with the online tool‐based ceRNA network, an HCC‐specific ceRNA network was obtained, and lncRNA‐miRNA‐mRNA regulatory axes were extracted. RNAs associated with prognosis were further obtained, and multivariate Cox regression models were established to identify the prognostic signature and nomogram. As a result, 198 DElncRNAs, 120 DEmiRNAs, and 2827 DEmRNAs were identified, and 30 key genes identified from the differential network were enriched in four cancer‐related pathways. Four HCC‐specific lncRNA‐miRNA‐mRNA regulatory axes were extracted, and SNHG11, CRNDE, MYLK‐AS1, E2F3, and CHEK1 were found to be related with HCC prognosis. Multivariate Cox regression analysis identified a prognostic signature, comprised of CRNDE, MYLK‐AS1, and CHEK1, for overall survival (OS) of HCC. A nomogram comprising the prognostic signature and pathological stage was established and showed some net clinical benefits. The AUC of the prognostic signature and nomogram for 1‐year, 3‐year, and 5‐year survival was 0.777 (0.657‐0.865), 0.722 (0.640‐0.848), and 0.630 (0.528‐0.823), and 0.751 (0.664‐0.870), 0.773 (0.707‐0.849), and 0.734 (0.638‐0.845), respectively. These results provided clues for the study of potential biomarkers and therapeutic targets for HCC. In addition, the obtained 30 key genes and 4 regulatory axes might also help elucidate the underlying mechanism of HCC.

## Graphical abstract

This article constructed an HCC‐specific ceRNA network by integrating a differential lncRNA‐miRNA‐mRNA regulatory network and an online tool‐based ceRNA network. As a result, four lncRNA–miRNA–mRNA regulatory axes were extracted, and a prognostic signature (CRNDE, MYLK‐AS1 and CHEK1) was identified for overall survival (OS) of HCC. A nomogram comprising the prognostic signature and pathological stage was established and showed some net clinical benefits.

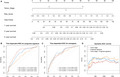

## 1 INTRODUCTION

Liver cancers are the fourth leading cause of cancer‐related death worldwide with a dismal 5‐year survival rate.^1^ Hepatocellular carcinoma (HCC) is the major type of primary liver cancers. It typically occurs in patients with underlying liver injury, which is mostly caused by hepatitis B or C virus infection or alcohol abuse. An effective prognostic assessment of HCC can be conducive to cancer management and clinical treatment. Currently, the evaluation of HCC prognosis is based mainly on tumor stage, histological grade, and serum alpha‐fetoprotein. However, considering the heterogeneity of HCC, the clinical outcomes of different individuals may vary greatly. Even among patients with the same clinicopathological characteristics, predicting the prognosis of HCC only on the basis of clinicopathological features may be finite. Consequently, more effective prognostic markers at the molecular level are needed. In addition, knowing that HCC is among the solid cancers with the fewest somatic mutations that can be targeted with molecular therapies,^2^ it is vital to take molecular markers into account when identifying the future promising predictive biomarkers and/or potential therapeutic targets.

Long noncoding RNAs (lncRNAs) are important regulators of various biological processes, including abnormal transcriptional regulation in tumors, such as cell proliferation, cell apoptosis, and cell cycle regulation.^3^ A key regulatory mechanism for lncRNA is the competitive endogenous RNA (ceRNA) hypothesis, that is, ceRNA binds to microRNA (miRNA) through microRNA response elements and affects microRNA‐induced gene silencing.^4^ LncRNA has been suggested as a potential therapeutic target and biomarker in many diseases due to its conserved secondary structure and higher tissue and organ specificity.^5^ Recently, several studies have emerged, in which the cancer prognostic signature was identified by the analysis of a dysregulated lncRNA‐related ceRNA regulatory network. For HCC, Bai et al.^6^ constructed a risk score system based on 13 lncRNAs through ceRNA network analysis. Liao et al.^7^ constructed a prognostic signature using three lncRNAs and six mRNAs by establishing a ceRNA network. Zhang et al.^8^ identified eight mRNA biomarkers through the ceRNA network and constructed a predictive model for overall HCC survival. However, at present, the construction of a ceRNA network is mainly based on nondisease‐specific online tools, a majority of which are based on binary ceRNA pair. Thus, despite being simple and convenient, these studies based on online tools might not be reflective of the specific population under study and have failed to consider the complex interactions among genes. In contrast, gene‐regulatory network analysis could provide a natural framework to explore the interactions among genes and take full account of disease‐specific data characteristics. In addition, exploring the topological changes in disparate biological groups (i.e., differential network analysis) may yield new findings and be conducive to the exploration of underlying mechanisms of diseases.^9^ Graphical models are common methods of constructing differential networks, which can eliminate many indirect and spurious associations through conditional independent tests so as to obtain a reliable network. However, due to the background noise, some false positives may still exist in the data‐driven methods.^10^ Considering this, integrating the data‐driven reconstructed network and the prior‐driven network from online tools may be a desirable alternative to obtain a robust disease‐specific biologically regulatory network.

In this study, a graphical‐model‐based approach, which has not been applied in ceRNA network construction, was used to construct a differential lncRNA‐miRNA‐mRNA network with TCGA‐LIHC data. By using the new strategy of integrating differential network with online tool‐based ceRNA network, a robust and reliable HCC‐specific lncRNA‐miRNA‐mRNA network was obtained. Then, the HCC‐specific regulatory axes were extracted, from which, the prognostic signature was further identified. The new strategy in this study could provide powerful guidance in elucidating lncRNA‐mediated underlying regulatory mechanisms and identifying potential biomarkers or therapeutic targets for HCC. Additionally, the prognostic signature identified from the regulatory axes was of more biological significance.

## 2 METHODS

**Figure 1:**
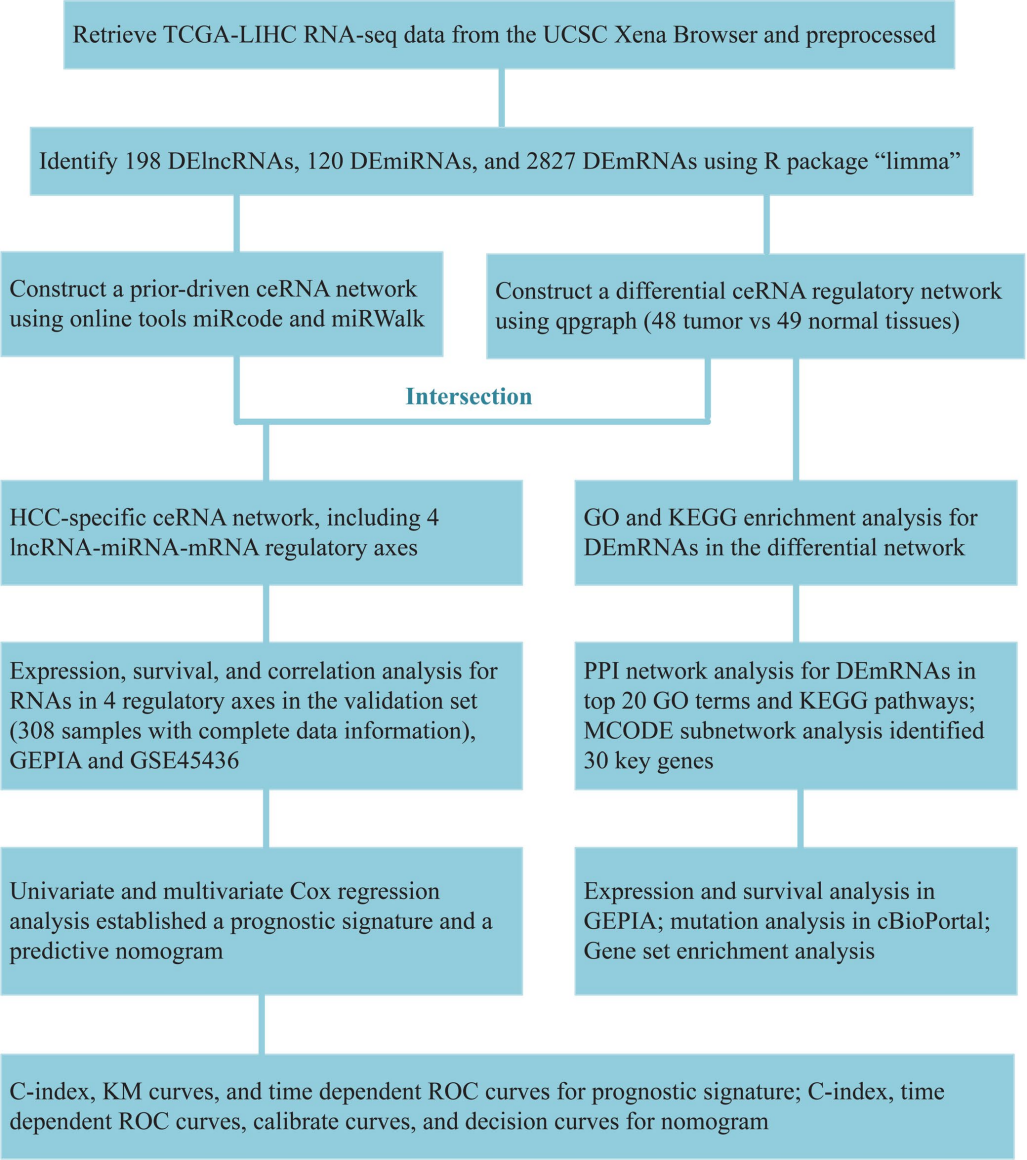
Workflow of the present study

The workflow of the present study is displayed in Figure 1.

### 2.1 RNA‐Seq data collection and preprocessing

The gene expression RNA‐seq data of the GDC TCGA‐LIHC cohort, miRNA mature strand expression RNA‐seq data of the TCGA‐LIHC cohort, and the corresponding phenotype data were retrieved from UCSC Xena Browser (https://xenabrowser.net) on April 7, 2020. The gene expression profiles of 374 HCC and 50 adjacent normal liver tissues and the miRNA data of 371 HCC and 49 adjacent normal liver tissues were included in the present study. LncRNAs and mRNAs in the expression matrix were annotated and distinguished according to the GTF file (gencode.v33.annotation.gtf) from GENCODE (https://www.gencodegenes.org/ ). The raw log2 (FPKM +1) RNA expression data were transformed into transcripts per million (TPM) using the following formula:

$${\text{TPM}}_{i}=\left(\frac{{\text{FPKM}}_{i}}{\sum_{j}{\text{FPKM}}_{j}}\right)\cdot 10^{6}$$

### 2.2 Identification of differentially expressed RNAs

After excluding RNAs with missing or zero expression values in more than 80% of samples, differential expression analysis was performed between tumor and normal tissues using the R package limma. RNAs with |log2 fold change (FC)| >1 and adjusted *P* value <0.05 were considered differentially expressed. The corresponding volcano plots and heatmaps were visualized using the R package ggplot2 and pheatmap, respectively.

### 2.3 Construction of a differential lncRNA‐miRNA‐mRNA network with qpgraph

The expression matrixes of DElncRNAs, DEmiRNAs, and DEmRNAs were merged by sample ID, and samples that failed to match were excluded. Then, 49 normal tissues and the corresponding 48 tumor tissues were selected to construct a lncRNA‐miRNA‐mRNA triple network for the normal and tumor groups, respectively. The edges that did not overlap in the two networks were selected to construct the differential network, which was named as data‐driven ceRNA network. The remaining samples in the merged RNA data set (i.e., excluding those used to construct the differential network) were matched with the corresponding phenotype data and served as a validation set for further analysis; duplicate samples and those with incomplete survival information were deleted.

LncRNA‐miRNA‐mRNA interactive networks were constructed with R/Bioconductor package qpgraph (available at http://www.bioconductor.org). Qpgraph was a comprehensive network analysis method by means of q‐order correlation graph. By performing conditional independence tests of order *q*, many indirect or spurious relationships could be removed. Another advantage of qpgraph was that we can choose to calculate and output only the edges of interest, thus, saving a lot of time and computing space.^11^ In qpgraph, the associations between two vertices were measured using the non‐rejection rate (NRR), which was defined as the probability of not rejecting the null hypothesis of conditional independent tests in a limited number of uniformly randomly selected subsets Q, such as 100.^12^ In this study, *q* was assumed as 1, 10, 15, and 20, and the average NRR was calculated, whose threshold was defined as 0.05. In addition, only lncRNA‐miRNA interactions and miRNA‐mRNA interactions were calculated and output.

### 2.4 Functional enrichment and PPI network analyses

GO and KEGG pathway enrichment analyses were performed for DEmRNAs in the differential network with R/Bioconductor package clusterProfiler. GO terms and KEGG pathways with a *P* value <0.01 and a *q* value <0.05 were considered as significantly enriched. Genes in the top 20 significant biological process (BP) terms, cellular component (CC) terms, molecular function (MF) terms, and all the KEGG pathways were filtered to perform PPI network analysis with an online STRING database (https://string‐db.org/). Only experiments or co‐expression gene pairs with a combined score ≥0.9 (the highest confidence) were included in the PPI network. Then, the MCODE algorithm was applied to identify the densely connected module using Cytoscape plugin MCODE with the threshold parameters degree cutoff =4, node score cutoff =0.6, K‐core =4, and maximum depth =100. The key genes in the highly correlated subnetworks were identified using a Cytoscape plugin CytoNCA with centrality analyses methods. The expression and survival analyses of the key genes were further performed in the GEPIA database (http://gepia.cancer‐pku.cn/).^13^ The gene mutation analysis was performed in cBioPortal (https://www.cbioportal.org/). To explore the functions and potential molecular mechanisms underlying the identified key genes, gene set enrichment analysis (GSEA) was performed using the clusterProfiler package. After performing 1000 permutations, gene sets with a *P* value <0.05 and a *q* value <0.05 were considered significantly enriched.

### 2.5 Construction of a ceRNA network with online tools

The identified DElncRNAs, DEmiRNAs, and DEmRNAs were used to construct a ceRNA network based on the hypothesis that lncRNAs affected the regulation of miRNAs on mRNAs by playing a sponge‐like role. The lncRNA‐miRNA interactions were predicted using miRcode (http://www.mircode.org). The target mRNAs of miRNAs were predicted using miRWalk (http://mirwalk.umm.uni‐heidelberg.de/ ). Only mRNAs predicted by three databases miRDB, Targetscan, and miRTarBase together were considered as miRNA targets. Then, lncRNA‐miRNA interactions and miRNA‐mRNA interactions sharing the same miRNAs were selected to construct a ceRNA network, which was named as a prior‐driven ceRNA network.

### 2.6 Identification and validation of HCC‐specific ceRNA network

The intersection of prior‐driven and data‐driven ceRNA networks was taken as the HCC‐specific ceRNA network, which was defined as “hub network” and visualized with R package igraph. The expressions, prognostic values and associations of interested RNAs in the hub network were evaluated in the validation set, GEPIA database and GEO dataset GSE45436 using Kaplan‐Meier (KM) estimate, log‐rank (LR) test, Spearman correlation analysis, and Wilcoxon rank sum test.

### 2.7 Establishment of the prognostic signature and nomogram

Multivariate Cox regression analysis was performed on DERNAs with prognostic values to obtain the prognostic signature for HCC using R package survival. Then, the risk score of every patient was calculated using regression coefficients, and the samples were divided into high‐ and low‐risk groups with the median risk score as a cutoff. The Kaplan‐Meier estimate and LR test were further performed to compare the survival time between the two groups. The C‐index and time‐dependent ROC curve were used to evaluate the predictive value of the prognostic signature. Subsequently, univariate and multivariate Cox regression analyses were performed on the prognostic signature along with the clinical variables. A nomogram was constructed with significant clinical characteristics and risk scores for better prediction and more convenient clinical application. The clinical and prognostic values of the nomogram were evaluated with C‐index, time‐dependent ROC curve, calibration curve, and decision curve analysis using R package survival, survivalROC, timeROC, rms, and stdca.R function.

## 3 RESULTS

### 3.1 Identification of DElncRNA, DEmiRNA, and DEmRNA in HCC

**Figure 2:**
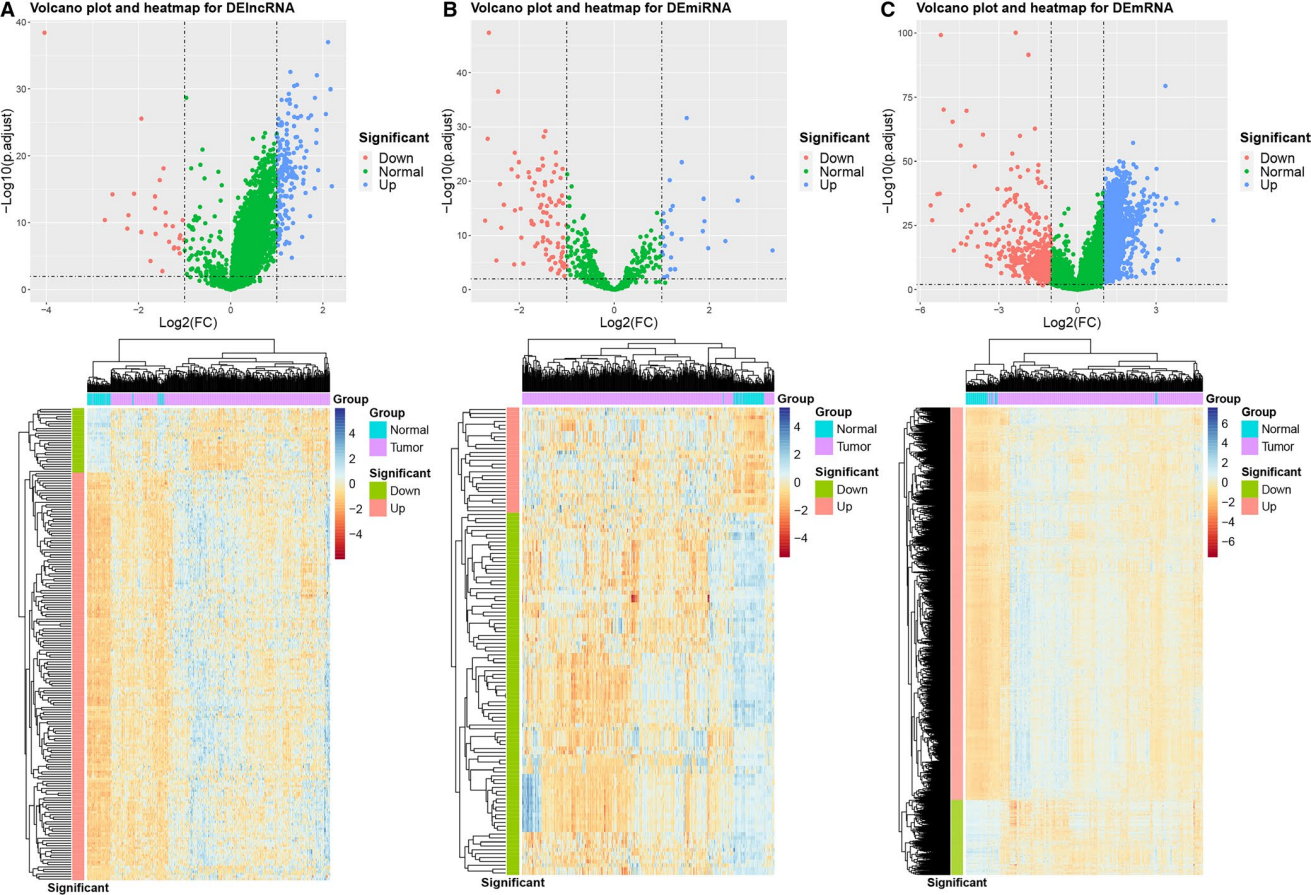
Volcano plots and heatmaps for the DERNAs. (A) DElncRNA (B) DEmiRNA (C) DEmRNA

According to the differential expression analysis, 198 DElncRNAs (171 upregulated and 27 downregulated), 120 DEmiRNAs (27 upregulated and 93 downregulated), and 2827 DEmRNAs (2375 upregulated and 452 downregulated) were identified between the tumor and adjacent normal tissues. The distributions and expression patterns of the DERNAs were displayed using volcano plots and heatmaps in Figure 2A‐Figure 2C.

### 3.2 Construction of a differential ceRNA network and identification of key genes

All the DERNAs were further used to construct a lncRNA‐miRNA‐mRNA regulatory network in the tumor and normal groups using qpgraph. With the conditional independence tests of order *q* (*q* = 1, 10, 15, and 20) and average NRR cutoff of 0.05, 11,024, and 4616 edges were identified in the tumor and normal groups, respectively. Then, a differential network containing 2654 vertices (194 DElncRNAs, 120 DEmiRNAs, and 2340 DEmRNAs) was constructed with the 15,018 non‐overlapping edges between the two groups (Fig. S1).

**Figure 3:**
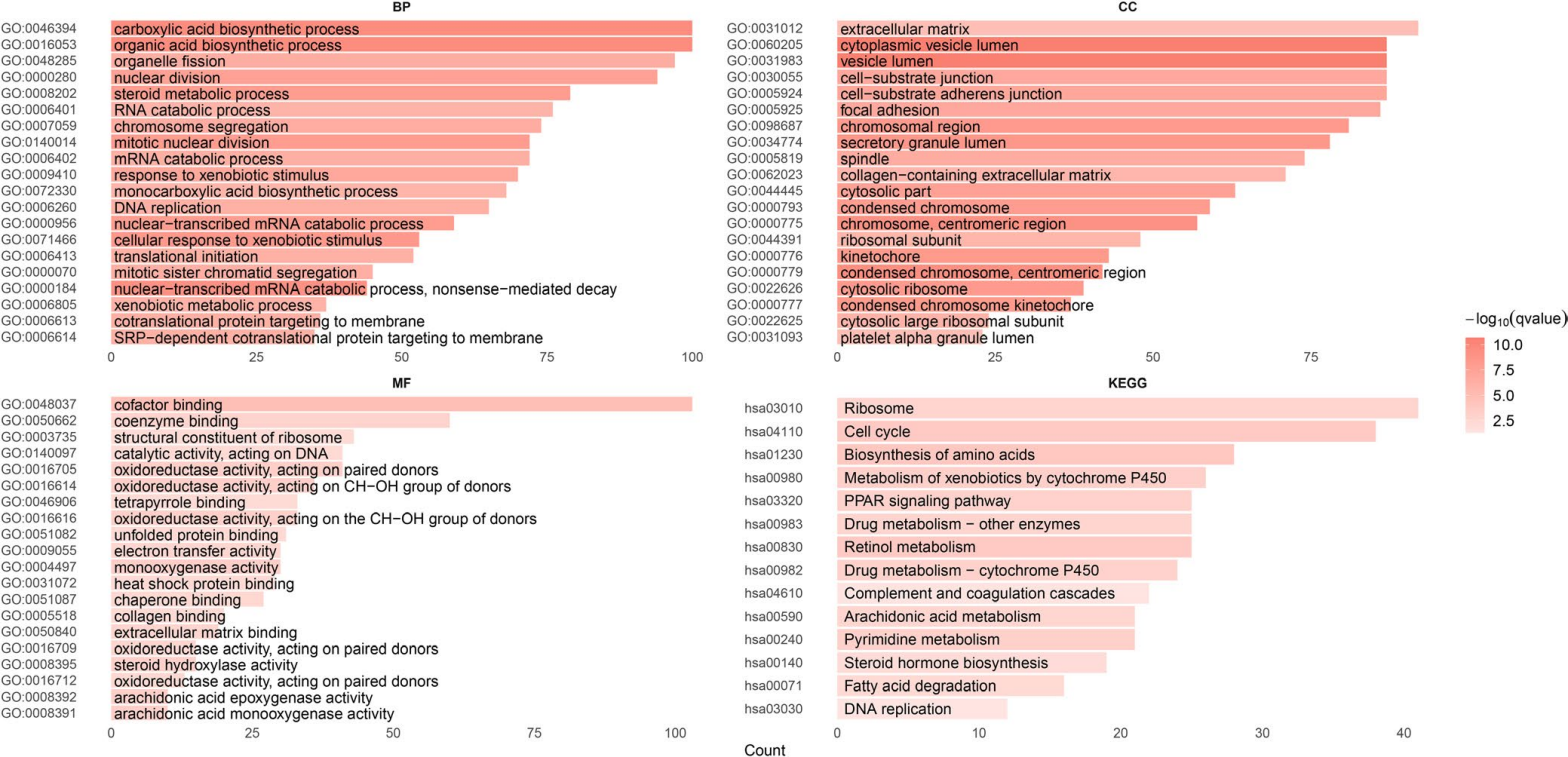
Top 20 GO terms (BP, CC, MF) and KEGG pathways

GO and KEGG enrichment analyses were performed on DEmRNAs involved in the network to explore the biological functions of the constructed differential network. With the threshold *P* value <0.01 and *q* value<0.05, 358 GO terms (282 BPs, 53 CCs, and 23 MFs) and 14 KEGG pathways were significantly enriched. The most significant BP, CC, MF, and KEGG pathway were carboxylic acid biosynthetic process (GO: 0046394, *p* = 9.53e ‐ 14), cytoplasmic vesicle lumen (GO: 0060205, *p* = 5.58e ‐ 14), cofactor binding (GO: 0048037, *p* = 1.49e ‐ 08), and biosynthesis of amino acids (hsa01230, *p* = 7.03e ‐ 07), respectively. Figure 3 displays the top 20 GO terms and KEGG pathways, among which 864 DEmRNAs were used to perform PPI network analysis and 1104 edges were output with a PPI enrichment *P* value <1.0e ‐ 16, under the threshold of a combined score ≥0.9. Then, MCODE module analysis identified four significantly correlated modules, and the top two most significant modules were used for the subsequent analysis, in which module 1 (Score =35.611) contained 37 nodes and 641 edges (Fig. S2), and module 2 (Score=11.111) contained 28 nodes and 150 edges (Fig. S3). Next, 19 genes in module 1 and 11 genes in module 2 were identified as key genes according to the centrality analysis because they ranked at the top in the list of all the eight centralities (Subgraph, Degree, Eigenvector, Information, LAC, Betweenness, Closeness, and Network). They were all upregulated in HCC tumor tissues, and most of them were shown to be associated with the overall survival and were differentially expressed between tumor and normal tissues (matched TCGA normal and GTEx data) according to the survival and expression analysis in GEPIA (Table 1). The mutation analysis in cBioPortal showed that 178 (48.63%) of the 366 TCGA‐LIHC patients presented with alterations in the 30 genes, and amplification constituted the major mutation type (Fig. S4). GSEA was further performed to explore the functions and potential molecular mechanisms of the 30 key genes. The results showed that they were enriched in four KEGG pathways: Ribosome (hsa03010, *p* = 0.0056), progesterone‐mediated oocyte maturation (hsa04914, *p* = 0.0100), cellular senescence (hsa04218, *p* = 0.0259), and cell cycle (hsa04110, *p* = 0.0315) (Table A1).

**Table 1:** Expression and Survival analysis of key genes

Gene	log2 *FC*	Adjust *P* value	GEPIA2 (Match TCGA normal and GTEx data)		
			Log‐rank *P* value	*HR* (high)	Significance between T&N
Module 1					
RPL10A	1.004600	1.16E−17	0.0500	1.4	‐‐
RPL23	1.064366	4.84E−19	0.0069	1.6	*
RPL23A	1.153929	2.62E−18	0.0011	1.8	*
RPL27	1.158706	2.18E−22	0.0017	1.8	*
RPL30	1.324543	2.85E−24	0.2100	1.2	*
RPL35A	1.080957	2.18E−20	0.0210	1.5	*
RPL36	1.018820	2.42E−15	0.0700	1.4	*
RPL37	1.111796	1.63E−20	0.0330	1.5	*
RPL38	1.306653	1.35E−23	0.0094	1.6	*
RPL8	1.478085	2.17E−21	0.0087	1.6	*
RPS16	1.132487	8.09E−20	0.0330	1.5	*
RPS18	1.191214	6.06E−19	0.0780	1.4	*
RPS2	1.037972	3.96E−16	0.0520	1.4	‐‐
RPS21	1.342644	2.01E−20	0.0370	1.4	*
RPS27	1.200106	7.01E−20	0.0670	1.4	*
RPS27A	1.032179	4.03E−18	0.0021	1.7	‐‐
RPS3	1.049939	1.98E−16	0.0110	1.6	‐‐
RPS7	1.173015	4.07E−24	0.0360	1.5	*
RPS8	1.012328	2.64E−17	0.0050	1.6	‐‐
Module 2					
ASPM	1.946575	6.67E−26	0.0006	1.8	*
AURKA	2.542411	9.91E−40	0.0002	1.9	*
AURKB	2.368675	1.76E−28	0.0280	1.5	*
BUB1B	1.707165	2.94E−21	0.0028	1.7	*
CCNA2	2.459235	8.96E−29	0.0037	1.7	*
CCNB1	2.911400	7.41E−38	0.0002	2.0	*
KIF20A	2.225952	2.04E−27	0.0034	1.7	*
MAD2L1	1.480848	5.16E−22	0.0047	1.7	*
NUSAP1	2.297342	1.90E−28	0.0063	1.6	*
TPX2	2.152649	2.70E−22	0.0005	1.9	*
UBE2C	3.373763	2.77E−36	0.0530	1.4	*

* Indicated that *p* < 0.05 between tumor and normal tissues.

### 3.3 Construction of a Prior‐driven ceRNA network

For DElncRNAs, 729 lncRNA‐miRNA interactions were predicted by miRcode; for DEmiRNAs and DEmRNAs, 273 miRNA‐mRNA interactions were predicted by miRWalk. In total, 255 lncRNA‐miRNA pairs and 247 miRNA‐mRNA pairs sharing the common miRNAs were selected to construct a prior‐driven ceRNA network, which included 27 DElncRNAs, 30 DEmiRNAs, and 182 DEmRNAs (Fig. S5).

### 3.4 Construction and validation of an HCC‐specific ceRNA network

**Figure 4:**
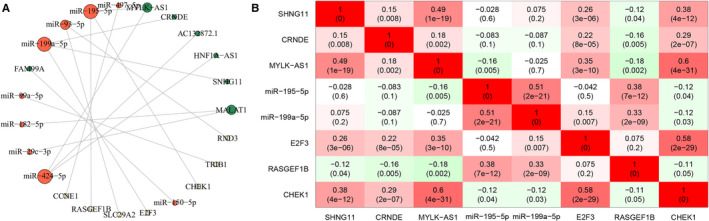
(A) Visualization of the HCC‐specific ceRNA network. The red, green and yellow nodes denoted miRNA, lncRNA, and mRNA, respectively. While size of the circles represented the node degrees. (B) Correlation heatmap for DERNAs in the four lncRNA–miRNA–mRNA regulatory axes. The texts in the grid denoted the Spearman correlation coefficients and the corresponding *P* values.

In order to elucidate the lncRNA‐miRNA‐mRNA regulatory mechanism in HCC, we take the intersection of the prior‐driven ceRNA network and the differential ceRNA regulatory network, and an HCC‐specific ceRNA network was obtained (Fig. 4A). This network consisted of 16 edges including nine lncRNA‐miRNA pairs and seven miRNA‐mRNA pairs. These comprised four lncRNA‐miRNA‐mRNA regulatory axes, namely SNHG11/hsa‐miR‐199a‐5p/E2F3, CRNDE/hsa‐miR‐199a‐5p/E2F3, MYLK‐AS1/hsa‐miR‐195‐5p/CHEK1, and MYLK‐AS1/hsa‐miR‐195‐5p/RASGEF1B. The eight DERNAs in the four regulatory axes were selected for further analysis. Of these, lncRNA SNHG11, CRNDE, and MYLK‐AS1 were upregulated, and miRNA miR‐199a‐5p and hsa‐miR‐195‐5p were downregulated. Further, mRNA E2F3 and CHEK1 were upregulated in the tumor group, which conformed to the ceRNA theory. However, mRNA RASGEF1B was downregulated in the tumor group, which deserves further investigation (Table 2).

**Table 2:** DERNAs in the four lncRNA‐miRNA‐mRNA regulatory axes

RNA	Log2 FC	*P* value	Adjust *P* value
lncRNA			
SNHG11	1.270008	9.28E−23	5.03E−21
CRNDE	1.572058	8.94E−18	1.38E−16
MYLK‐AS1	1.032068	2.67E−23	1.56E−21
miRNA			
has‐miR−195‐5p	−1.801820	7.72E−24	2.25E−22
has‐miR−199a−5p	−1.95506	4.28E−11	2.46E−10
mRNA			
E2F3	1.070448	5.75E−14	1.97E−13
CHEK1	1.259250	7.16E−22	5.18E−21
RASGEF1B	−1.112270	1.83E−15	7.17E−15

**Figure 5:**
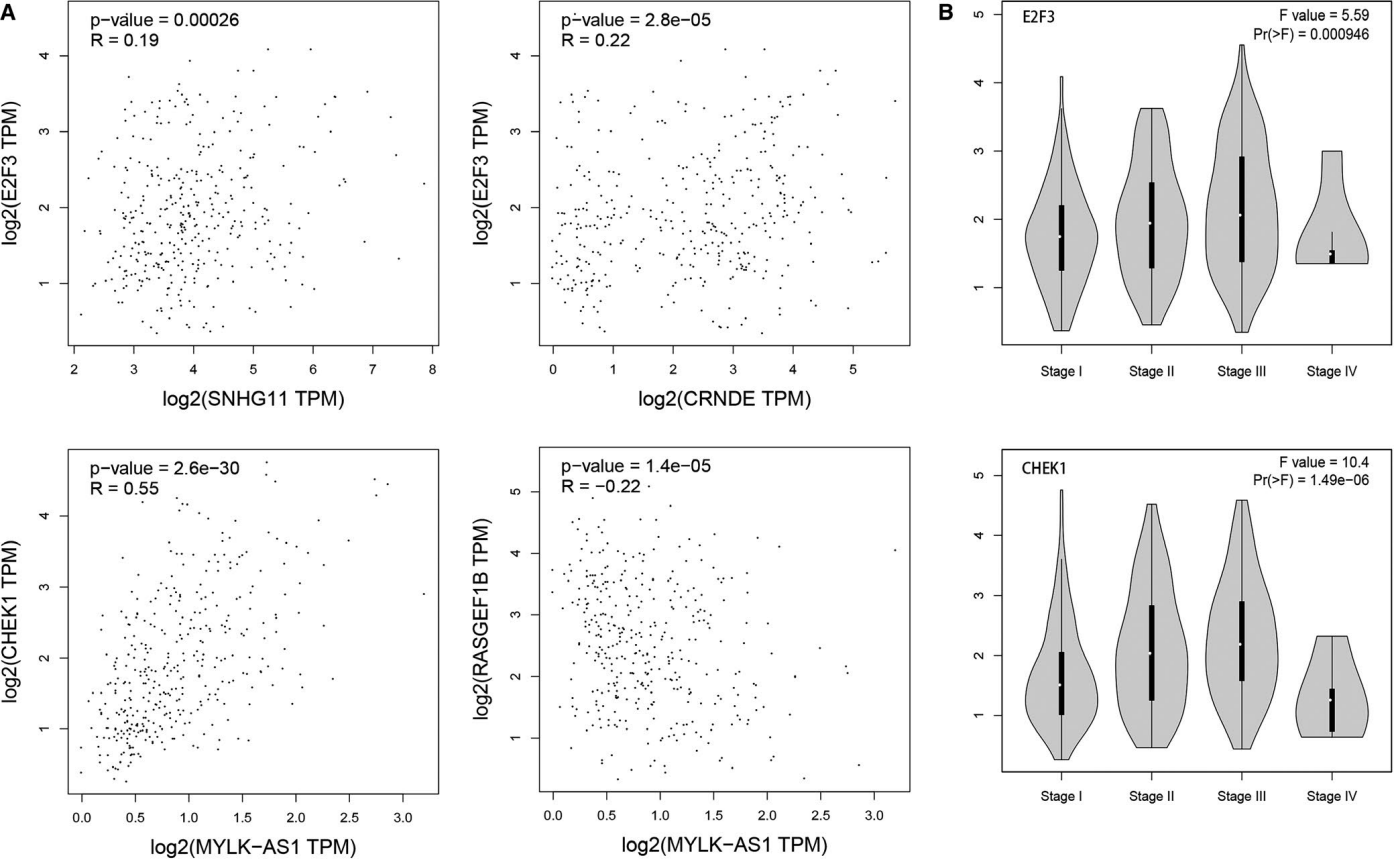
Validation of the eight DERNAs in the four regulatory axes with GEPIA LIHC tumor samples. (A) Scatter plots for Spearman correlation analysis. (B) Expression levels of E2F3 and CHEK1 in different tumor pathological stages

**Figure 6:**
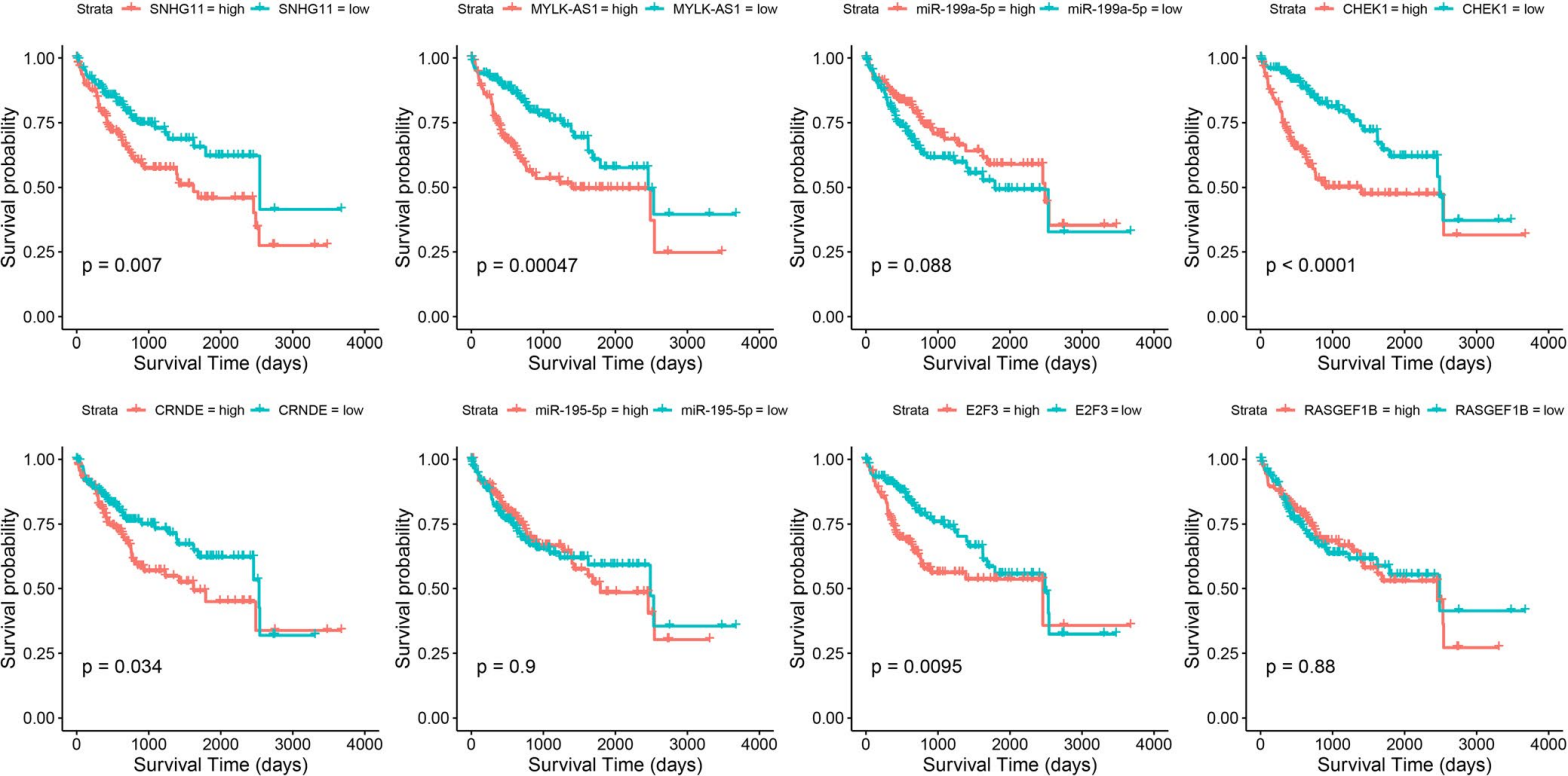
KM curves and log‐rank tests for the eight DERNAs in the validation set

Subsequently, Spearman correlation analysis among the eight DERNAs was performed in the validation set. The correlation heatmap in Figure 4B showed that SNHG11 and CRNDE positively correlated with E2F3; MYLK‐AS1 negatively correlated with miR‐195‐5p and RASGEF1B, and positively correlated with CHEK1. Further correlation analysis using data of HCC tumor samples in GEPIA and GEO dataset GSE45436 showed almost the consistent conclusions (Fig. 5A and Fig. S6). KM estimate and LR test in the validation set showed that high expression of SNHG11 [*HR*(high) =1.762, 95% *CI*: 1.161‐2.675], CRNDE [*HR*(high) =1.555, 95% *CI*: 1.032‐2.343], MYLK‐AS1 [*HR*(high) =2.079, 95% *CI*: 1.367‐3.162], E2F3 [*HR*(high) =1.716, 95% *CI*: 1.136‐2.593], and CHEK1 [*HR*(high) =2.568, 95% *CI*: 1.677‐3.931] were risk factors for overall survival time of HCC. Also, the survival time was significantly shorter in the high‐expression group than in the low‐expression group (Fig. 6). The survival analysis in GEPIA had roughly the same conclusions except SNHG11 (Fig. S7). The expression analysis among major pathological stages in GEPIA showed that E2F3 and CHEK1 had differential expression levels among four stages, both with lower expression levels in stage IV (Fig. 5B). In GSE45436, SNHG11, CRNDE, MYLK‐AS1, E2F3, and CHEK1 were highly expressed in the tumor group, while RASGEF1B was the opposite, which were consistent with the results of TCGA (Fig. S8).

### 3.5 Identification of prognostic signatures

**Figure 7:**
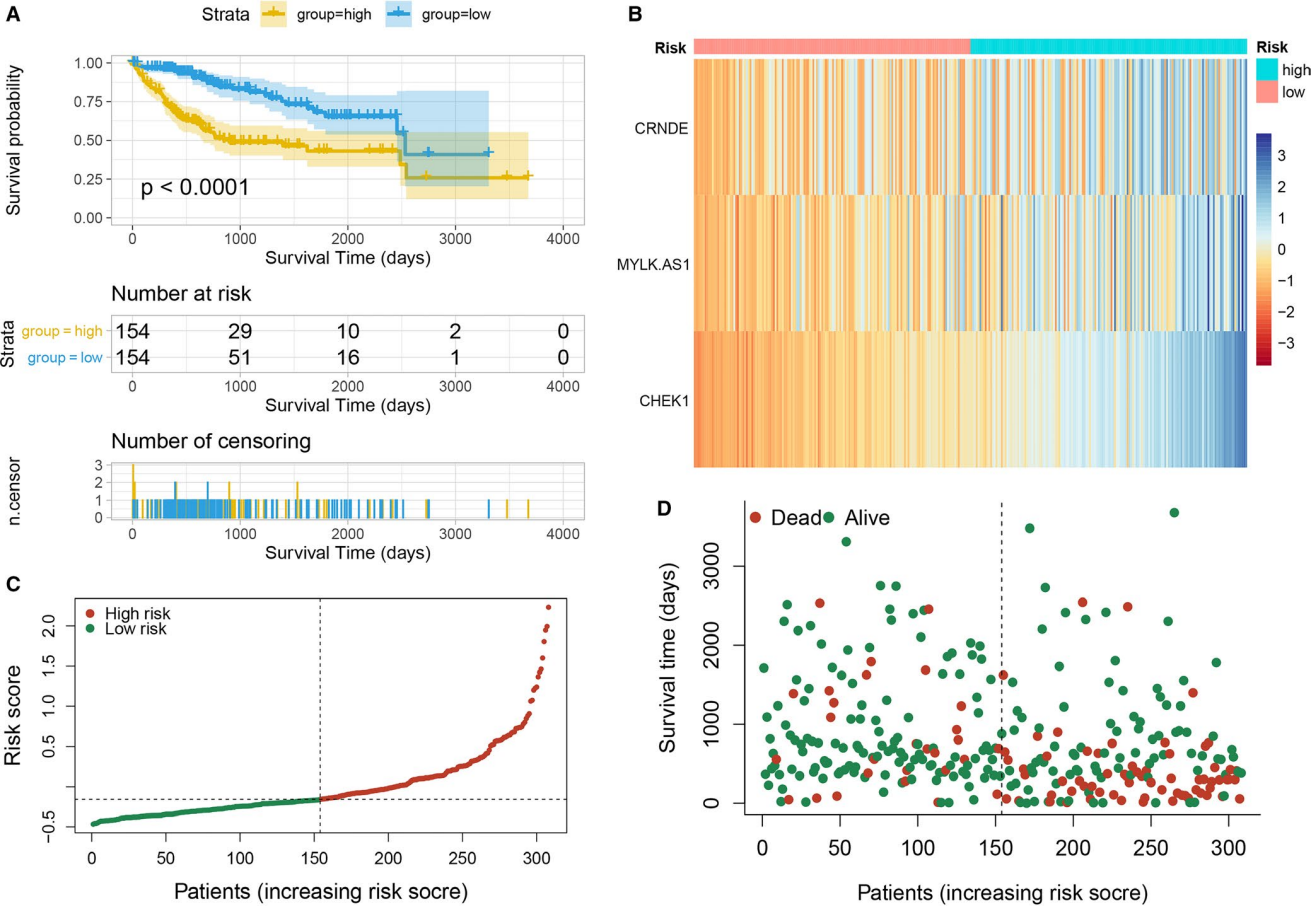
(A) KM curves and log‐rank test for patients in the high‐ and low‐ risk groups. (B) Risk heatmap of CRNDE, MYLK‐AS1 and CHEK1 for high‐ and low‐ risk groups. (C) Risk curve. (D) Survival state diagram

In order to construct the prognostic model of HCC, lncRNA SNHG11, CRNDE, and MYLK‐AS1, and mRNA E2F3 and CHEK1, which were significantly associated with the prognosis of HCC, were selected to perform multivariate Cox regression with the optimal subset strategy. The final model consisting of CRNDE, MYLK‐AS1, and CHEK1 was established and served as a prognostic predictive model of HCC [C‐index =0.705 (95% *CI*: 0.650 to 0.760), *p* = 1e‐07]. The risk score of every patient was calculated using the following formula: risk score =0.02083 × Exp(CRNDE) +0.02074 × Exp(MYLK‐AS1) +0.10721 × Exp(CHEK1), where “Exp” denoted the expression level of lncRNA normalized by mean. Then, patients were further divided into high‐ and low‐risk groups according to the median risk score. The KM survival curves demonstrated that patients in the high‐risk group had significantly poorer outcomes compared with those in the low‐risk group [*HR* =3.199 (95%CI: 2.052 to 4.986), *p* = 6e‐08] (Fig. 7A). The time‐dependent ROC curve analysis showed that the AUC and 95% CI (calculated by 10‐fold cross validation) of 1‐year, 3‐year, and 5‐year survival predicted by the prognostic signature (risk score) was 0.777 (95%CI: 0.657‐0.865), 0.722 (95%CI: 0.640‐0.848), and 0.630 (95%CI: 0.528‐0.823), respectively, indicating its effectiveness as a potential prognostic biomarker (Fig. 9B). The risk heatmap, risk curve and survival state diagram for high‐ and low‐ risk groups were displayed in Figure 7B, Figure 7C and Figure 7D, respectively.

**Figure 8:**
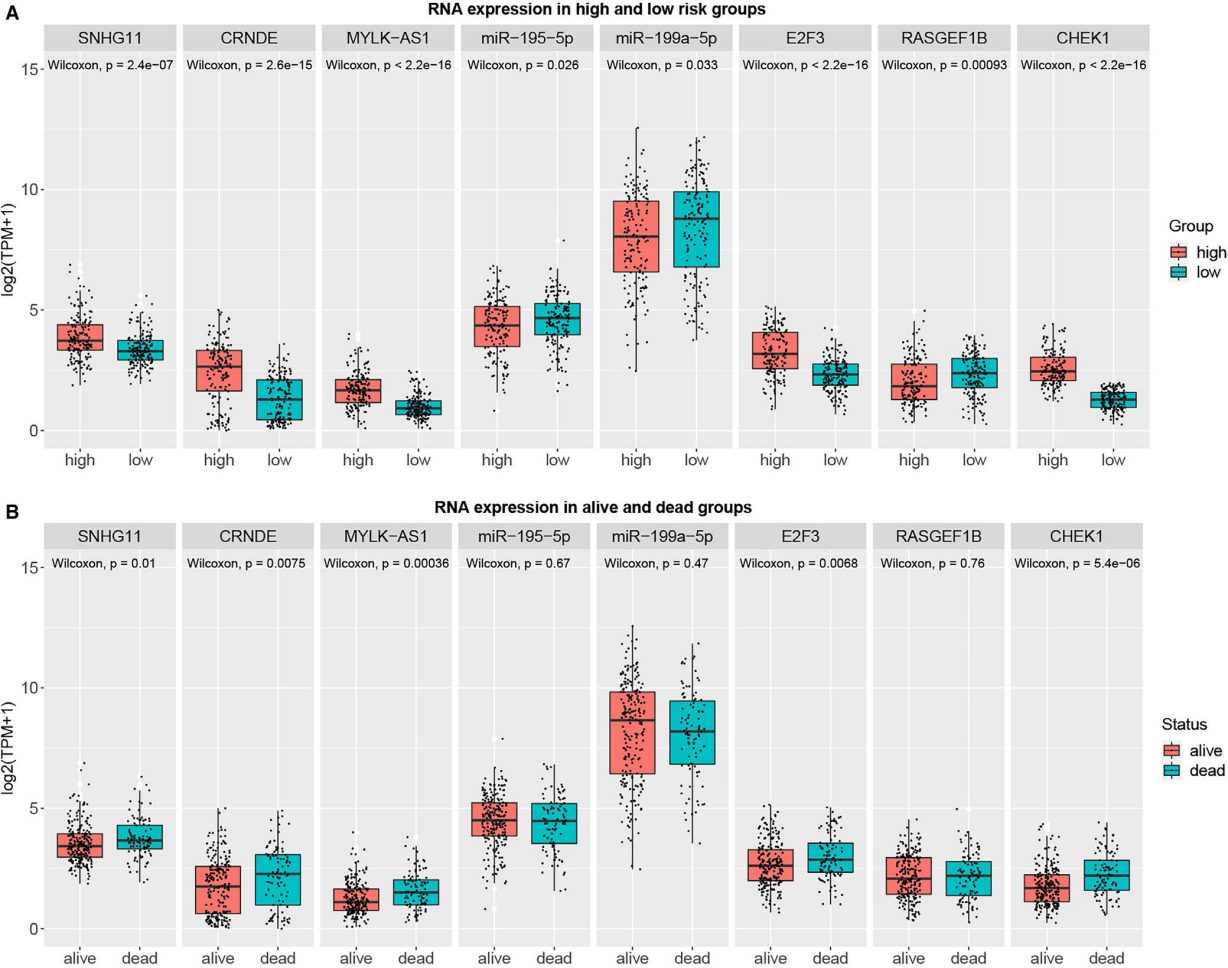
Expressions of the eight DERNAs in (A) high‐ and low‐risk groups, and (B) dead and alive groups

Subsequently, the expression levels of the eight RNAs involved in the four regulatory axes between high‐ and low‐risk groups and between dead and alive groups were analyzed. The results showed that SNHG11, CRNDE, MYLK‐AS1, E2F3, and CHEK1 were significantly highly expressed in the high‐risk and dead groups; while miR‐195‐5p, miR‐199a‐5p, and RASGEF1B were lowly expressed in the high‐risk group, with no significant difference between dead and alive groups (Fig. 8A and 8B).

### 3.6 Construction and evaluation of predictive nomogram

**Figure 9:**
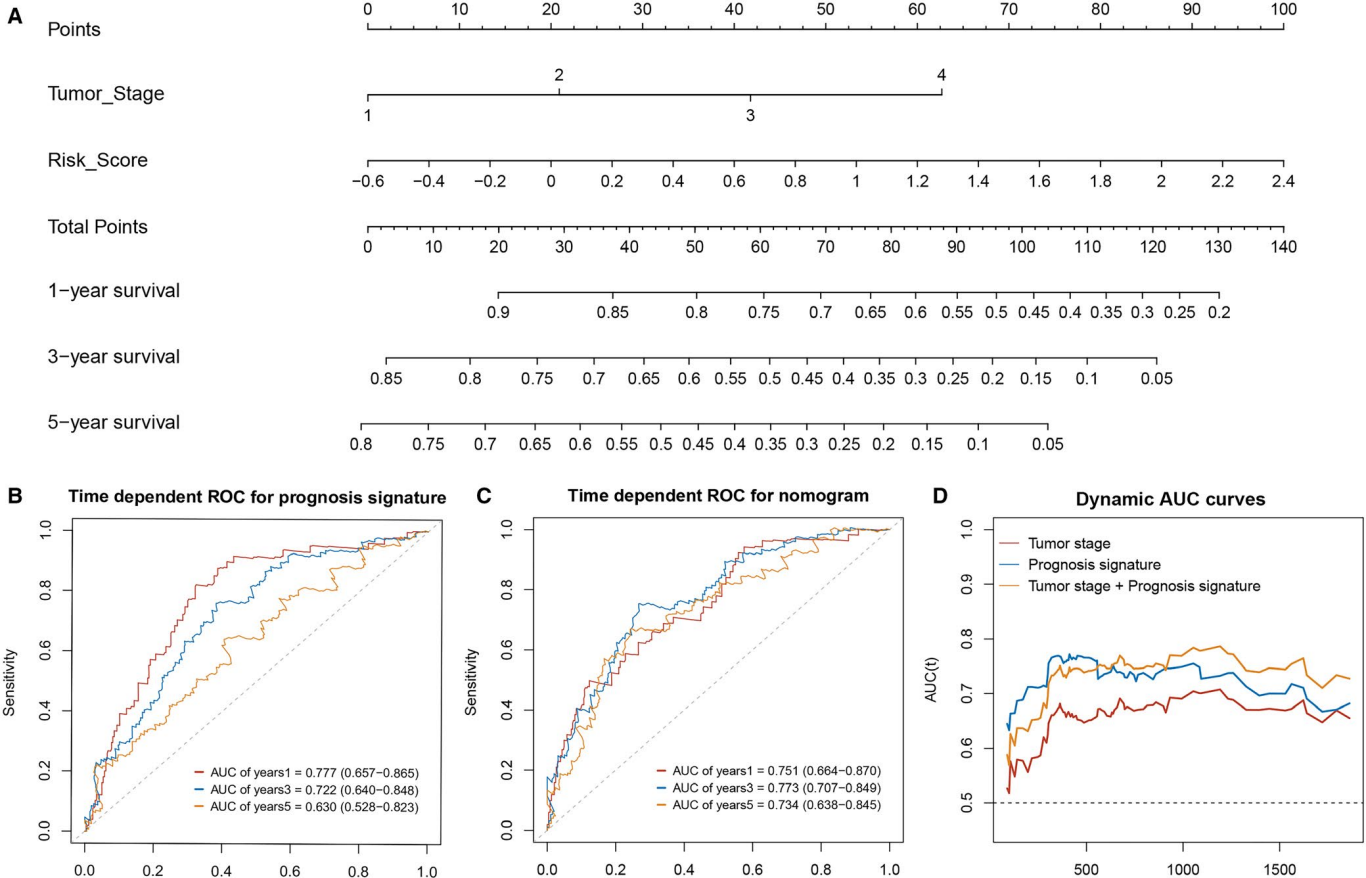
(A) Nomogram constructed by the prognosis signature (risk score) and tumor pathological stage. (B) Time dependent ROC curves of 1‐year, 3‐year and 5‐year survival for the prognosis signature. (C) Time dependent ROC curves of 1‐year, 3‐year and 5‐year survival for the nomogram. (D) Dynamic AUC curves for the single indicators and nomogram.

**Figure 10:**
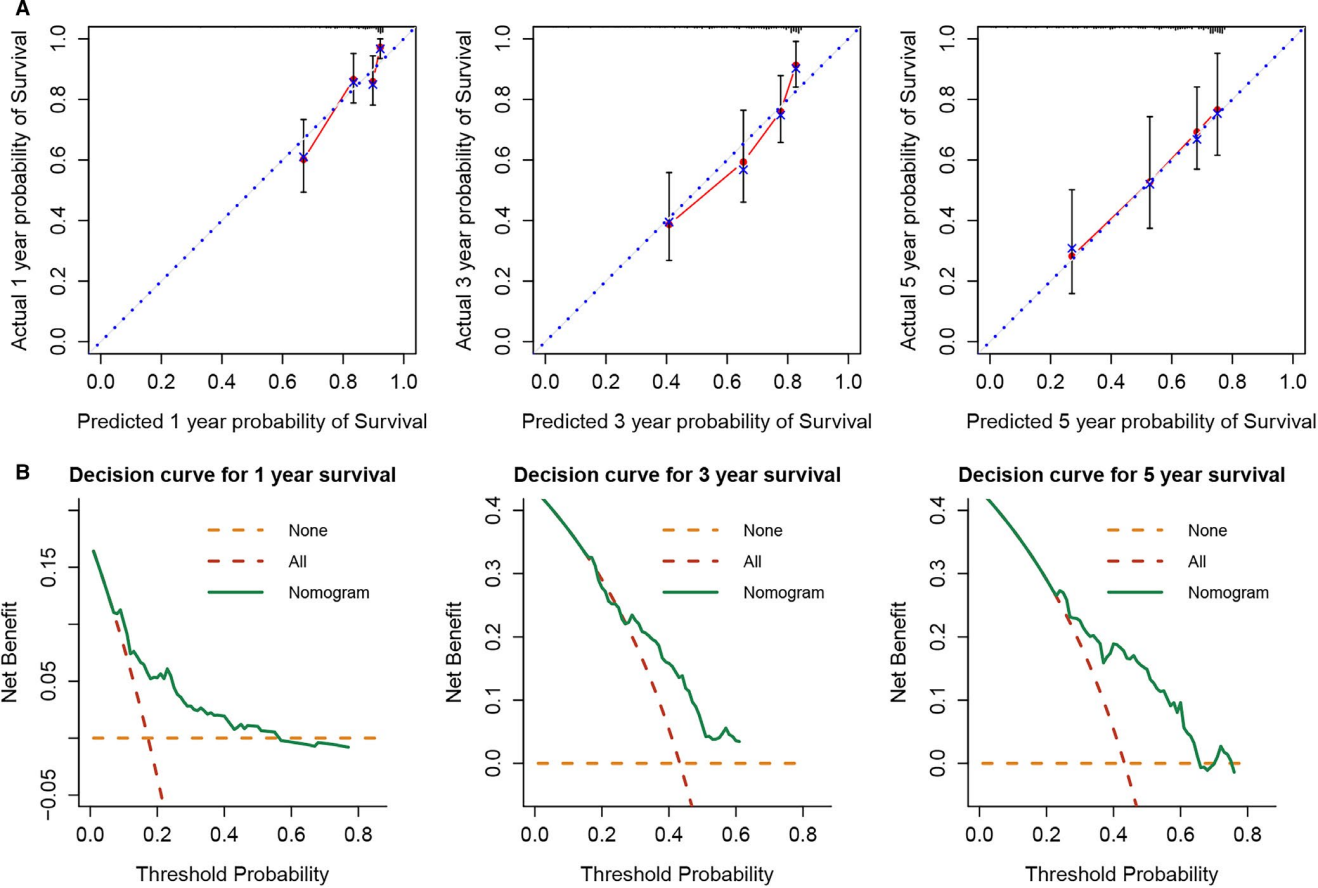
(A) Calibrate curves and (B) decision curves for the nomogram

Univariate and multivariate Cox regression analyses were performed for the clinical characteristics, including age, sex, body mass index (BMI), family history, pathological stage, and histological grade, along with the prognostic signature. The pathological stage and prognostic signature were found to be independent prognostic factors for the overall survival of HCC (Table 3). To gain the clinical practical value, a predictive nomogram was established and evaluated using the pathological stage and prognostic signature (Fig. 9A). The C‐index of the nomogram was 0.704 (95% CI: 0.647‐0.761), and the AUC and 95% CI (calculated by 10‐fold cross validation) of 1‐year, 3‐year, and 5‐year survival was 0.751 (95% CI: 0.664‐0.870), 0.773 (95% CI: 0.707‐0.849), and 0.734 (95% CI: 0.638‐0.845), respectively (Fig. 9C). The dynamic AUC curves of the pathological stage, prognostic signature, and nomogram demonstrated the better predictive power of the prognostic signature and nomogram compared with the pathological stage (Fig. 9D). The calibration curves for the 1‐year, 3‐year, and 5‐year survival probability showed good consistency between the predicted and actual observations (Fig. 10A). The decision curves of 1‐year, 3‐year, and 5‐year survival showed that within the threshold probability range of approximately 0.05‐0.6, 0.3‐0.6, and 0.25‐0.75, intervening in the patients would get more net benefit than the treat‐all‐ or treat‐none‐patients scheme (Fig. 10B).

**Table 3:** Univariate and multivariate Cox regression analysis of clinical characteristics and lncRNA signature on overall survival of HCC

Variable	Univariate analysis		Multivariate analysis	
	*HR* (95% CI)	*P* Value	*HR* (95% *CI*)	*P* Value
Age	1.019(1.002–1.037)	0.028		
Gender	1.381(0.903–2.112)	0.137		
BMI	1.003(0.969–1.038)	0.880		
Family history	0.945(0.599–1.490)	0.807		
Pathological Stage	1.816(1.443–2.286)	3.73E−07	1.677 (1.318–2.132)	2.51E−05
Histological Grade	1.144(0.880–1.487)	0.315		
prognosis signature	2.718(1.932–3.825)	9.52E−09	2.281 (1.580–3.293)	1.08E−05

## 4 DISCUSSION

HCC is a highly heterogeneous malignancy with a poor prognosis and remains a major public health hazard. An in‐depth exploration of the underlying mechanisms and identification of effective prognostic indicators for HCC are conducive to clinical treatment decisions and patient management. Recently, an increasing number of studies have illustrated that lncRNA plays critical regulatory roles in HCC through lncRNA‐miRNA‐mRNA axes. CACNA1G‐AS1 promoted the progression of HCC via competitively binding miR‐2392 and alleviating its inhibition on C1orf61.^14^ MCM3AP‐AS1 promoted the expression of FOXA1 through targeting miR‐194‐5p and thus exerted an oncogenic role in HCC.^15^ DSCR8 facilitated the activation of the Wnt/β‐catenin signaling pathway in HCC via the DSCR8/miR‐485‐5p/FZD7 axis.^16^ A few studies identified HCC‐related potential prognostic biomarkers via the construction of the ceRNA network.^6^, ^17^, ^18^ However, they were based on online tools that failed to take complex interactions among genes or disease‐specific data features into account. This problem was explored in the present study with a new strategy, and an HCC‐specific ceRNA network was constructed, from which the disease‐specific regulatory axes were extracted. Then, the prognostic signature and predictive nomogram were further established.

The differential ceRNA network was constructed using qpgraph, a network reconstruction method that makes full use of the disease‐specific data to analyze complex interactions among genes. In qpgraph, many indirect and spurious associations could be eliminated through conditional independent tests. Moreover, it allowed to calculate only interested edges so as to obtain a relatively concise and reliable network.

A total of 30 key genes were obtained from PPI network analysis for mRNAs in the top 20 GO terms and KEGG pathways of the data‐driven differential network. Among them, 18 ribosomal protein genes were enriched in the ribosomal pathway. Besides being important components of ribosomes, ribosomal proteins also performed some extra‐ribosomal functions, including oncogenic, tumor‐suppressive, and immune responses.^19^ For example, RPL8 participated in the immune response and promoted tumorigenesis by activating NF‐κB^b^;^20^ RPS3 acted as a tumor suppressor by activating p53, activating JNK through TRADD and cooperating with E2F1;^21^, ^22^, ^23^ and RPS7 played an anticancer role by activating p53, regulating PI3 K/Akt and MAPK, and stabilizing GADD45a.^24^, ^25^, ^26^ Besides, some ribosomal proteins performed both oncogenic and tumor‐suppressive roles. For instance, RPL23 functioned through sequestering NPM from Miz1,^27^ and RPS27 functioned through inducing ITGB4 and activating NF‐κB^b^ to inhibit tumors;^28^, ^29^ while both of them activated p53 to exert the anti‐cancer roles.^30^, ^31^ Importantly, RPL36 and RPS2 were reported to be overexpressed in HCC and performing the function of maintaining the synthetic function and facilitating cell proliferation, respectively.^32^, ^33^ Since tumor cells were more sensitive to ribosomal inhibition compared with normal somatic cells, and anti‐ribosome biogenesis drugs might be less genotoxic to normal cells,^19^, ^34^ these genes could be considered as potential candidates for the development of ribosomal targeted therapeutic strategies for HCC. Additionally, CCNB1 and CCNA2 were enriched in three interacting pathways: progesterone‐mediated oocyte maturation, cellular senescence, and cell cycle. Cyclin‐B‐Cdc2 kinase interacted with the mitogen‐activated protein (MAP) kinase cascade and participated in the regulation of cell cycle progression throughout the oocyte maturation.^35^ Cellular senescence was a highly stable cell cycle arrest, and the corresponding cell cycle exit was regulated by activating p53/p21^CIP1^ and p16^INK4a^/Rb tumor suppressor pathways.^36^ Intriguingly, the promoting effect of CCNB1 and CCNA2 on HCC has been reported in previous studies, whose high expression could promote cell proliferation, migration, and invasion and was closely related to the poor prognosis of HCC.^37^, ^38^ The important regulatory roles of CCNB1 and CCNA2 in HCC might provide new clues for exploring the underlying mechanism and potential therapeutic targets of HCC. In a word, the analyses about the 30 key genes helped to elucidate the underlying mechanisms and prognostic biomarkers for HCC from the perspective of biological pathways. Actually, it was also a combination of data information and biological priori information, which might provide powerful evidences for the further study.

Likewise, an HCC‐specific ceRNA network was constructed by integrating the prior‐driven and data‐driven ceRNA network, and four HCC‐related lncRNA‐miRNA‐mRNA regulatory axes SNHG11/hsa‐miR‐199a‐5p/E2F3, CRNDE/hsa‐miR‐199a‐5p/E2F3, MYLK‐AS1/hsa‐miR‐195‐5p/CHEK1, and MYLK‐AS1/hsa‐miR‐195‐5p/RASGEF1B were identified. Considering the potential advantages of molecular marker as a biomarker or therapeutic target, DERNAs with prognostic values in the four regulatory axes were further selected to construct a multivariate Cox regression model. Finally, CRNDE, MYLK‐AS1, and CHEK1 were identified as the prognostic signature, and patients were divided into high‐ and low‐risk groups with significantly different prognostic characteristics. The RNA expression levels in the four regulatory axes between high‐ and low‐risk groups were further analyzed. SNHG11, CRNDE, MYLK‐AS1, E2F3, and CHEK1 were found to be significantly highly expressed in the high‐risk group, while miR‐195‐5p, miR‐199a‐5p, and RASGEF1B were lowly expressed in the high‐risk group, demonstrating the vital roles of the four regulatory axes in the prognosis of HCC and the prognostic value of the prognostic signature.

SNHG11 was a member of a small nucleolar RNA host gene family involved in various tumor progressions, such as promoting proliferation and metastasis by targeting the Wnt/β‐catenin signaling pathway and the Hippo pathway in lung cancer and colorectal cancer, respectively.^39^, ^40^ In HCC, SNHG11 regulated proliferation, migration, apoptosis, and autophagy of cancer cells through a hsa‐miR‐184/AGO2 axis.^41^ Colorectal neoplasia differentially expressed (CRNDE) was a newly characterized oncogene whose levels were elevated in various human malignancies and associated with clinicopathological features and a poor prognosis, such as colorectal cancer, lung cancer, breast cancer, hepatocellular carcinoma, and so forth. It has been identified as a potential diagnostic and prognostic biomarker in various cancers due to the irreplaceable roles and the temporal and tissue‐specific expression patterns.^42^ CRNDE has been confirmed to promote HCC cell proliferation and growth through overexpression and knockdown experiments in vitro, and the oncogenic role was exerted by regulating PI3 K/Akt/β‐catenin signaling pathway.^43^ Besides, the oncogenic mechanism of CRNDE in HCC also included regulating the expression of NF‐κB and p‐Akt through suppressing miR‐384, as well as activating the mTOR signaling pathway by regulating the phosphorylation level of mTOR and P70S6 K,^44^, ^45^ etc. In the identified HCC‐specific ceRNA network, SNHG11 and CRNDE functioned in HCC through the hsa‐miR‐199a‐5p/E2F3 signaling pathway. MiR‐199a‐5p was shown to be downregulated and acted as a suppressor of the Warburg effect in HCC by targeting a 3’‐untranslated region (UTR) of hypoxia‐inducible factor‐1α (HIF‐1α) or hexokinase 2 (HK2), thereby suppressing glucose uptake and consumption, lactate production, and cell growth and proliferation.^46^ E2F3 was a member of the E2F transcription factor family, which regulated cellular proliferation and differentiation. The overexpression or amplification of E2F3 was common in various cancers, and its reduction could deter cancer progression.^47^ The copy number gains in E2F3b could induce spontaneous HCC in mice, and germ‐line loss protected mice against HCC.^48^ Therefore, it can be inferred that SNHG11/hsa‐miR‐199a‐5p/E2F3 and CRNDE/hsa‐miR‐199a‐5p/E2F3 signaling pathways might function through regulating cell proliferation and migration in HCC.

MYLK antisense RNA 1 (MYLK‐AS1) was found to be downregulated in colon adenocarcinoma and upregulated in HCC.^49^, ^50^ Recently, MYLK‐AS1 has been verified to promote HCC cell proliferation, migration, and invasion by overexpression and knockdown experiments in vitro. The mechanism was to regulate the EGFR/HER2‐ERK1/2 signaling pathway.^51^ In the present study, MYLK‐AS1 played roles in HCC by regulating CHEK1 and RASGEF1B through hsa‐miR‐195‐5p. MiR‐195 was reported to be downregulated in several cancers, and its overexpression could inhibit the proliferation and migration and regulate the G1/S transition of cancer cells in non‐small cell lung cancer, breast cancer, and hepatocellular carcinoma.^52^, ^53^, ^54^ In addition, miR‐195‐5p also acted as an anti‐oncogene via targeting PHF19 in HCC.^54^ CHEK1 was a serine/threonine‐specific protein kinase mediating cell cycle arrest in response to DNA damage and functioned through the PLK‐4/ATR/CHEK1 pathway in HCC.^55^, ^56^ RASGEF1B was a guanine‐nucleotide exchange factor(GEF) expressed in macrophages under the stimulation of Toll‐like receptor (TLR) agonists, and could mediate innate immune responses triggered during microbial infection.^57^ No mechanistic studies on RASGEF1B and HCC have been performed yet. Nevertheless, it is presumed that MYLK‐AS1/hsa‐miR‐195‐5p/CHEK1 and MYLK‐AS1/hsa‐miR‐195‐5p/RASGEF1B regulatory axes play important roles in HCC by regulating cell cycle and immune response.

The underlying functions and regulatory mechanisms of the aforementioned four regulatory axes indicated the potential value of CRNDE, MYLK‐AS1, and CHEK1 as prognostic markers. A combination of prognostic signature with conventional clinical characteristics might provide better predictive efficacy compared with a single biomarker. Hence, a nomogram was established in this study using the prognostic signature along with the tumor pathological stage, which also acted as an impact factor on the overall survival of HCC. The nomogram showed excellent performance in predicting the 1‐year, 3‐year, and 5‐year survival rates, and had a good clinical application value.

In summary, the identified 30 key genes functioned in HCC through four pathways: ribosomes, progesterone‐mediated oocyte maturation, cell senescence, and cell cycle. The four lncRNA‐miRNA‐mRNA regulatory axes might play vital roles in HCC by regulating cell proliferation, cell migration, cell cycle, and immune response. Importantly, CRNDE, MYLK‐AS1, and CHEK1 were identified as potential prognostic markers for HCC, and the corresponding nomogram showed some clinical net benefits. However, this study still had some limitations. First, the performance of the prognostic signature and nomogram could not be validated in external independent datasets due to either lack of MYLK‐AS1 expression data or lack of clinical and survival data. Second, the functions and regulatory mechanisms of the identified lncRNA‐miRNA‐mRNA axes in HCC need to be further verified by experimental studies.

## 5 CONFLICTS OF INTEREST

The authors declare that they have no conflicts of interest.

## ETHICS APPROVAL

No approval from the ethics committee was needed because all the information was required from the publicly available database.

6

## Supporting information

Fig S1

Fig S2

Fig S3

Fig S4

Fig S5

Fig S6

Fig S7

Fig S8

## Data Availability

All the data sets used in this study were publicly available at https://xenabrowser.net and http://www.ncbi.nlm.nih.gov/geo

## Appendix A

**Table A1:** GSEA KEGG pathways for key genes.

ID	Description	Size	ES	NES	p value	q value	Core enrichment
hsa03010	Ribosome	19	−1	−3.665	0.006	0.011	RPS21/RPL30/RPL38/RPS27/RPS18/RPS7/RPL27/RPL23A/RPS16/RPL37/RPL35A/RPL23/RPS3/RPS2/RPS27A/RPL36/RPS8/RPL10A
hsa04914	Progesterone‐mediated oocyte maturation	4	0.804	1.703	0.010	0.011	CCNB1/AURKA/CCNA2
hsa04218	Cellular senescence	2	0.929	1.584	0.026	0.017	CCNB1/CCNA2
hsa04110	Cell cycle	4	0.731	1.548	0.032	0.017	CCNB1/CCNA2/BUB1B/MAD2L1
