# Supplementary figures and images for: Construction of a disease‐specific lncRNA‐miRNA‐mRNA regulatory network reveals potential regulatory axes and prognostic biomarkers for hepatocellular carcinoma
Source: Cancer Med. 2020 Nov 24;9(24):9219–35. doi: 10.1002/cam4.3526 (PMC7774738; doi:10.1002/cam4.3526)

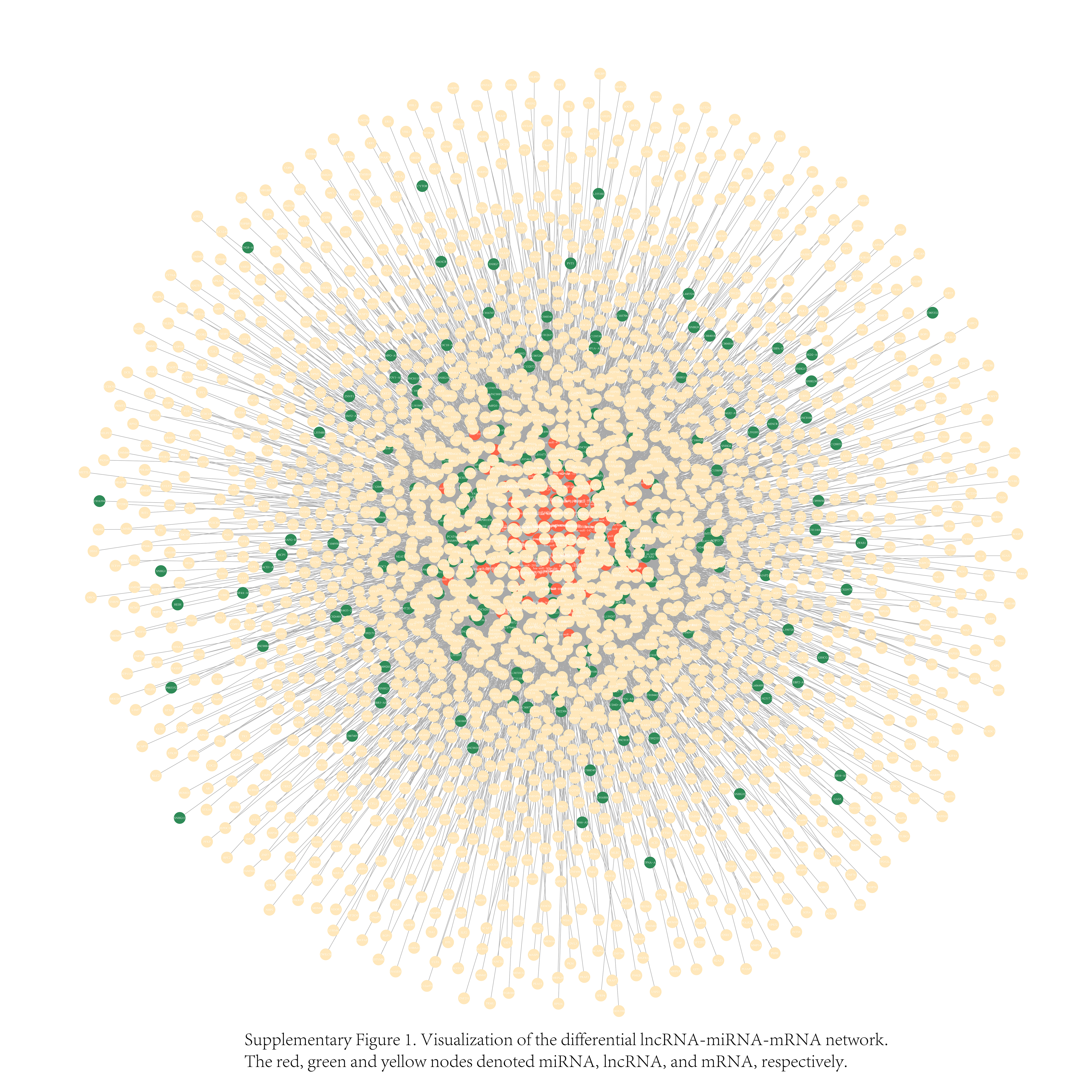

Supplement: Supplementary file 1 — Fig S1 [file CAM4-9-9219-s001.jpg]

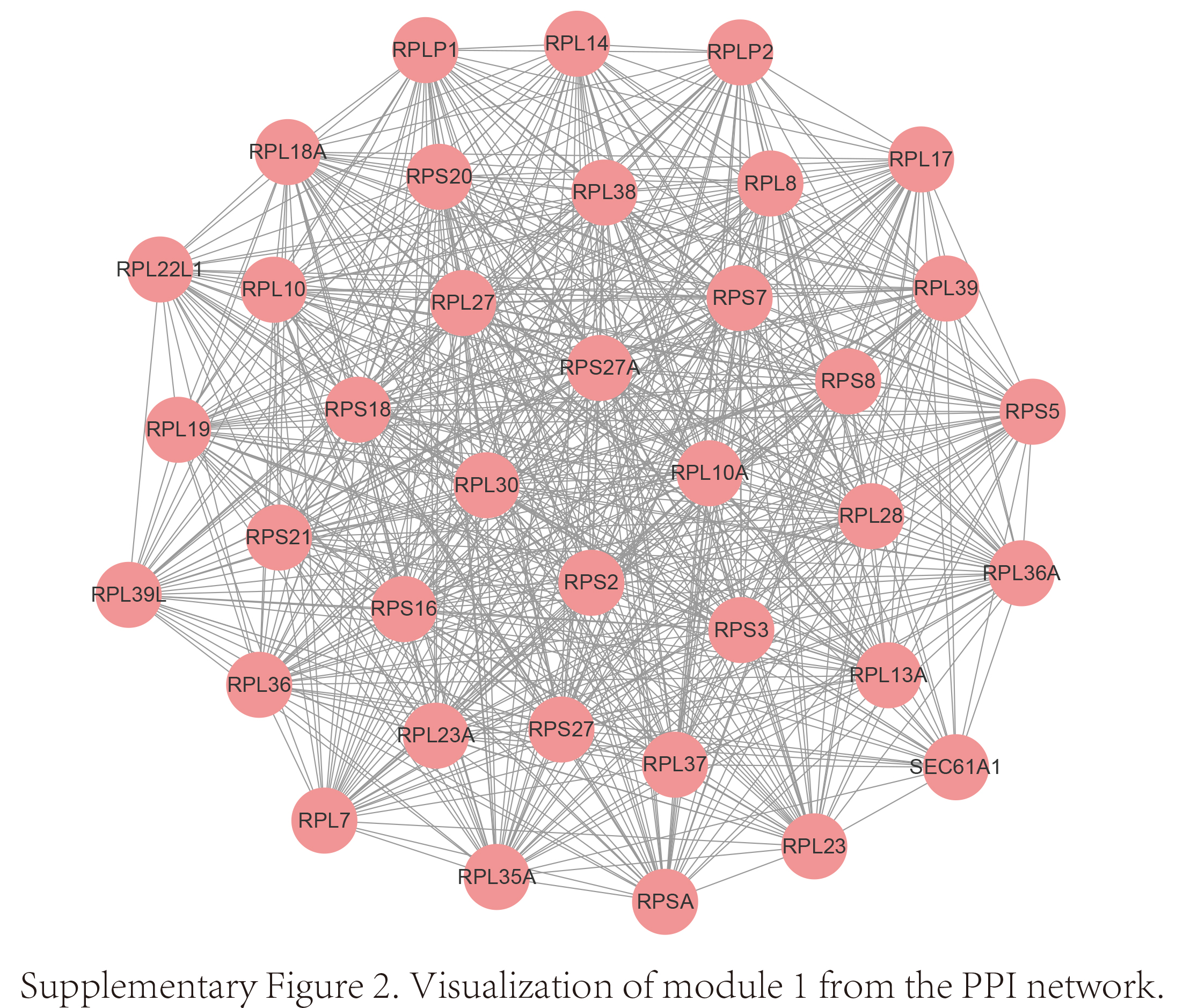

Supplement: Supplementary file 2 — Fig S2 [file CAM4-9-9219-s002.jpg]

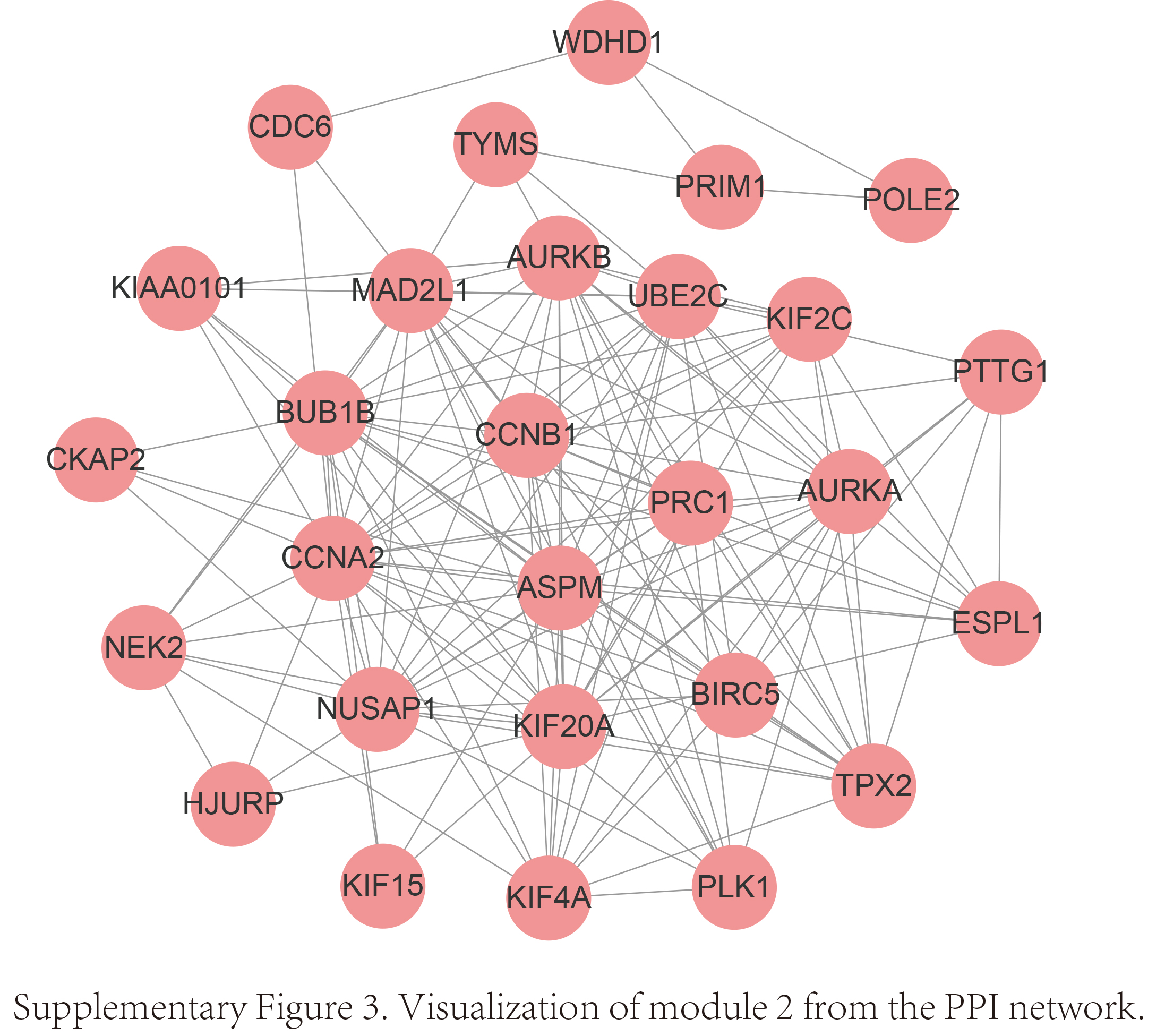

Supplement: Supplementary file 3 — Fig S3 [file CAM4-9-9219-s003.jpg]

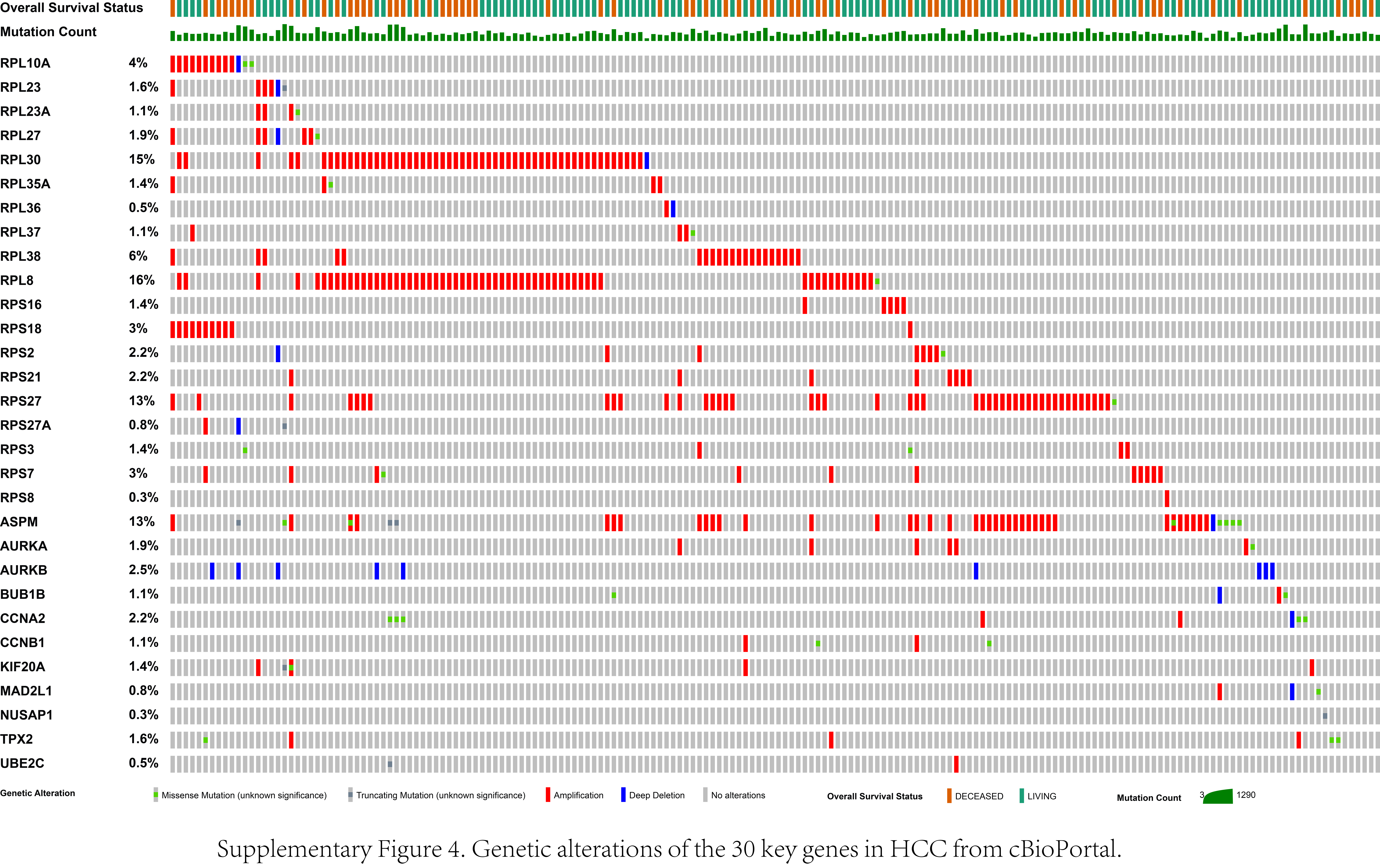

Supplement: Supplementary file 4 — Fig S4 [file CAM4-9-9219-s004.jpg]

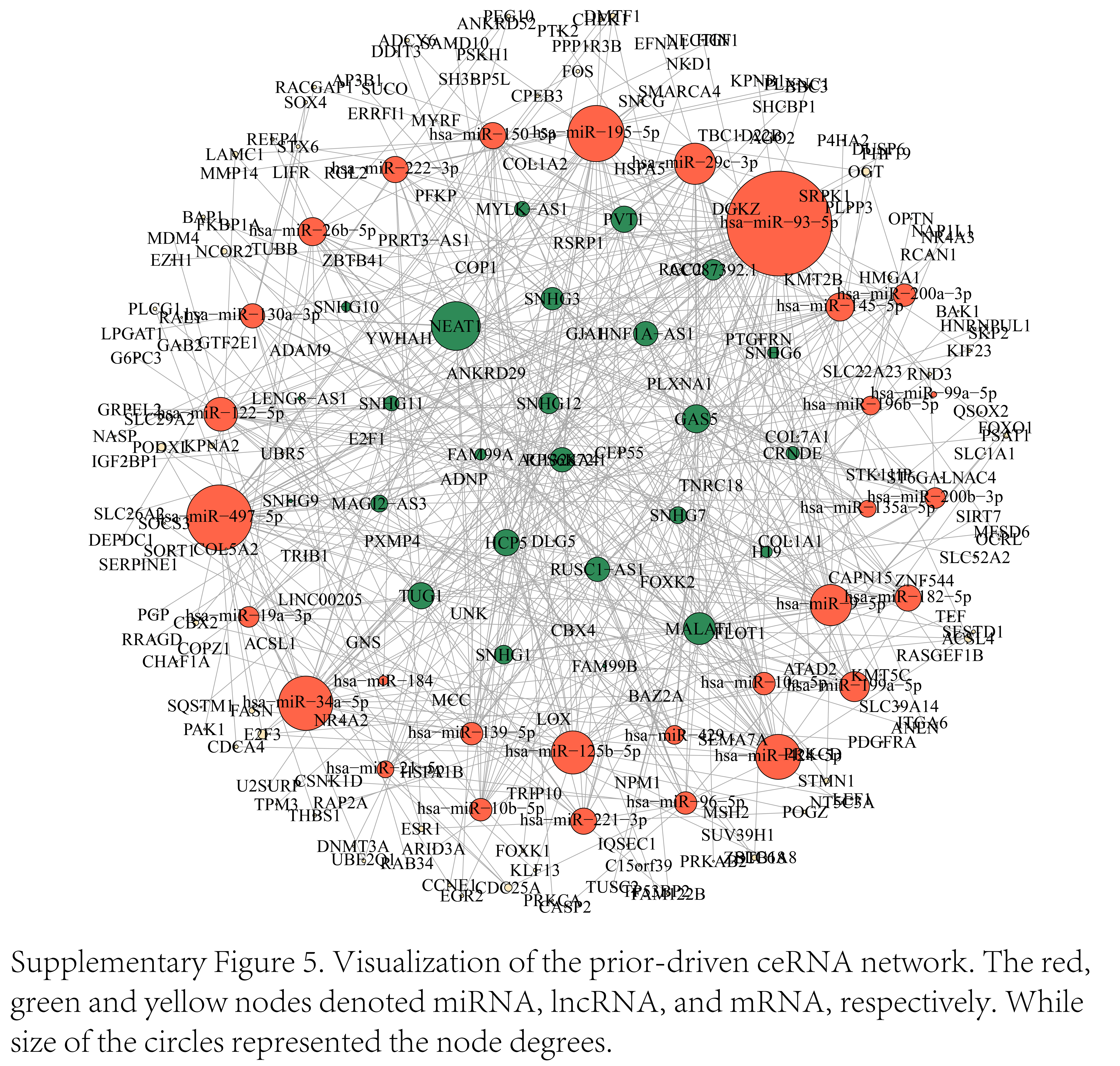

Supplement: Supplementary file 5 — Fig S5 [file CAM4-9-9219-s005.jpg]

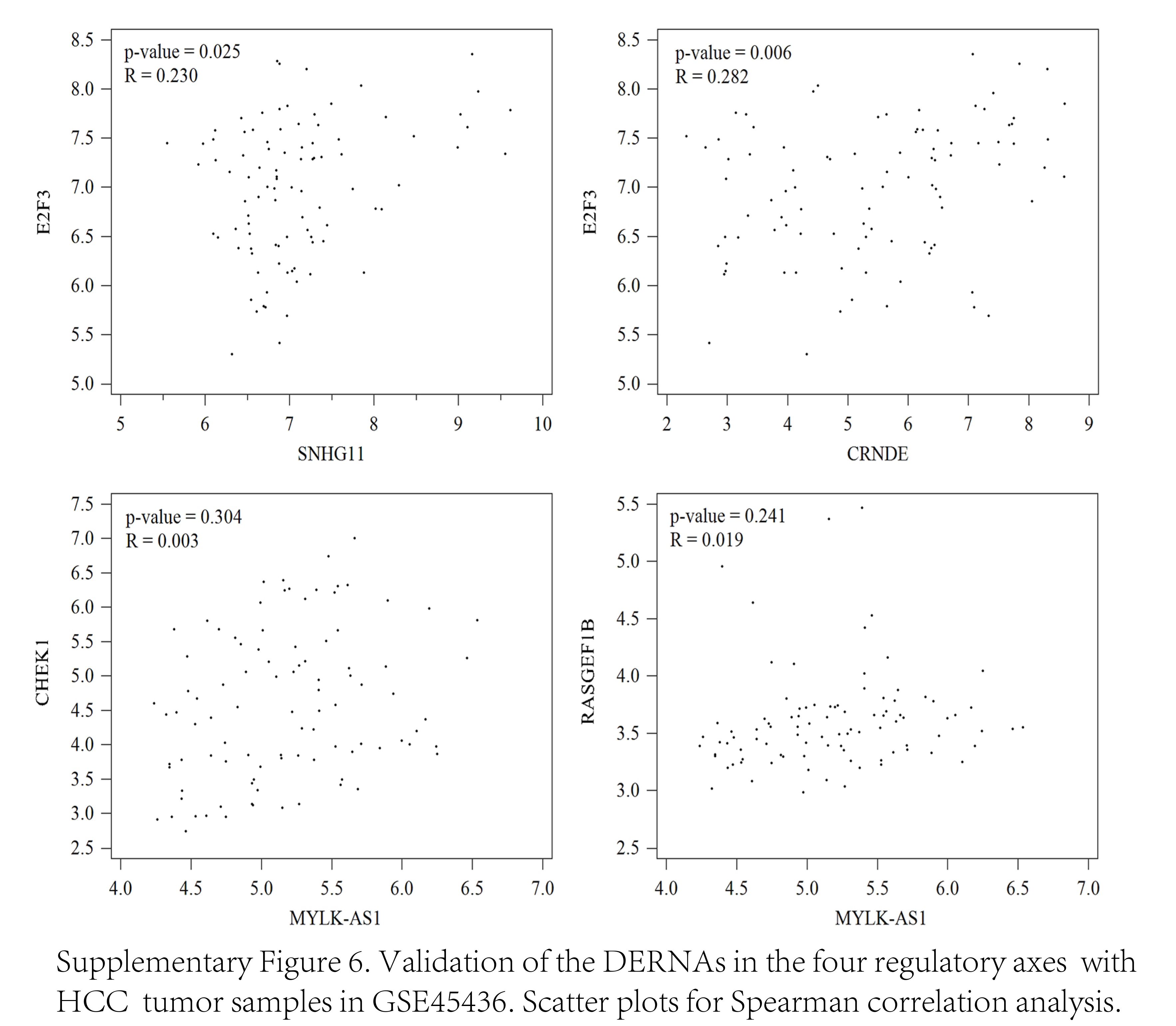

Supplement: Supplementary file 6 — Fig S6 [file CAM4-9-9219-s006.jpg]

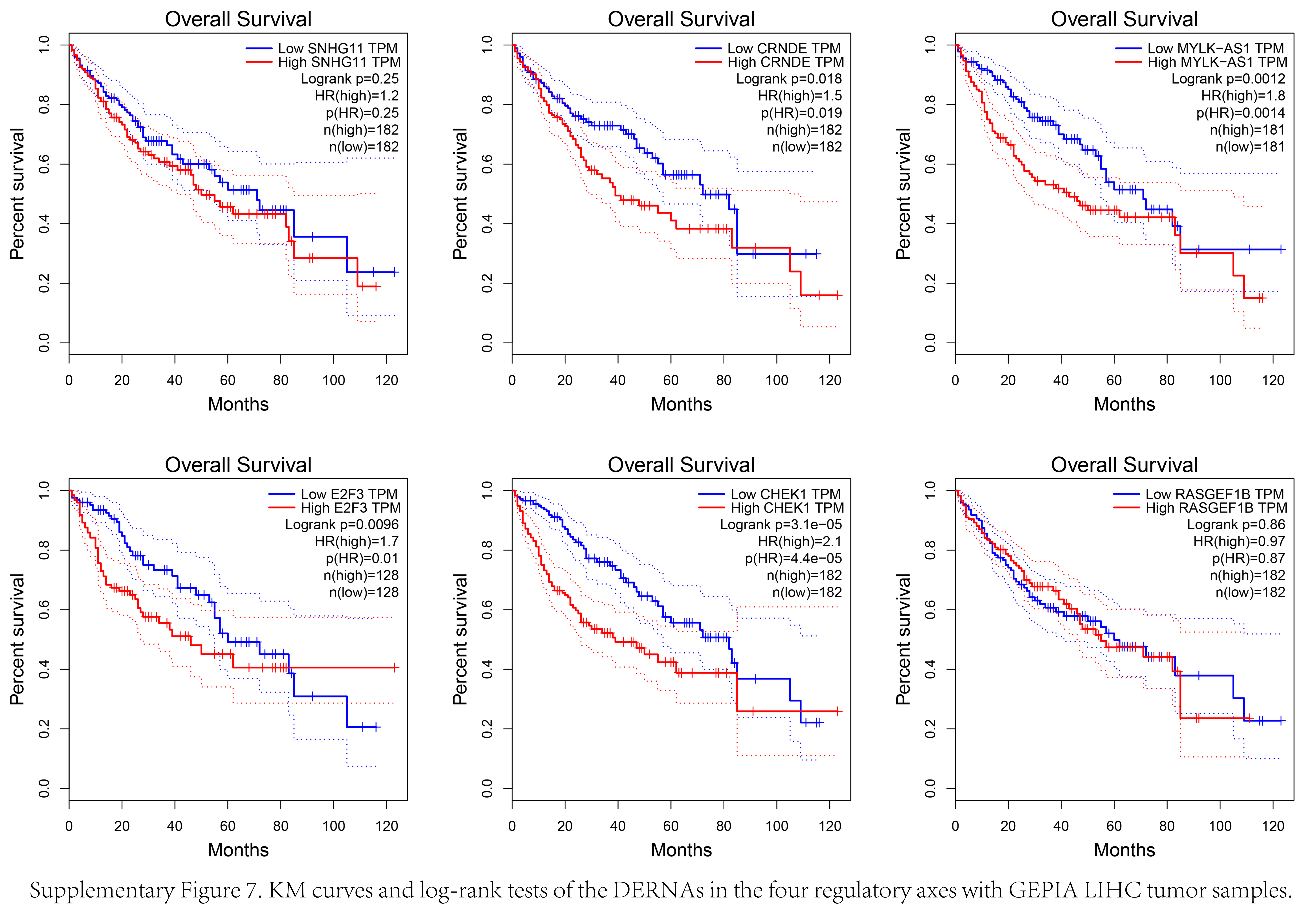

Supplement: Supplementary file 7 — Fig S7 [file CAM4-9-9219-s007.jpg]

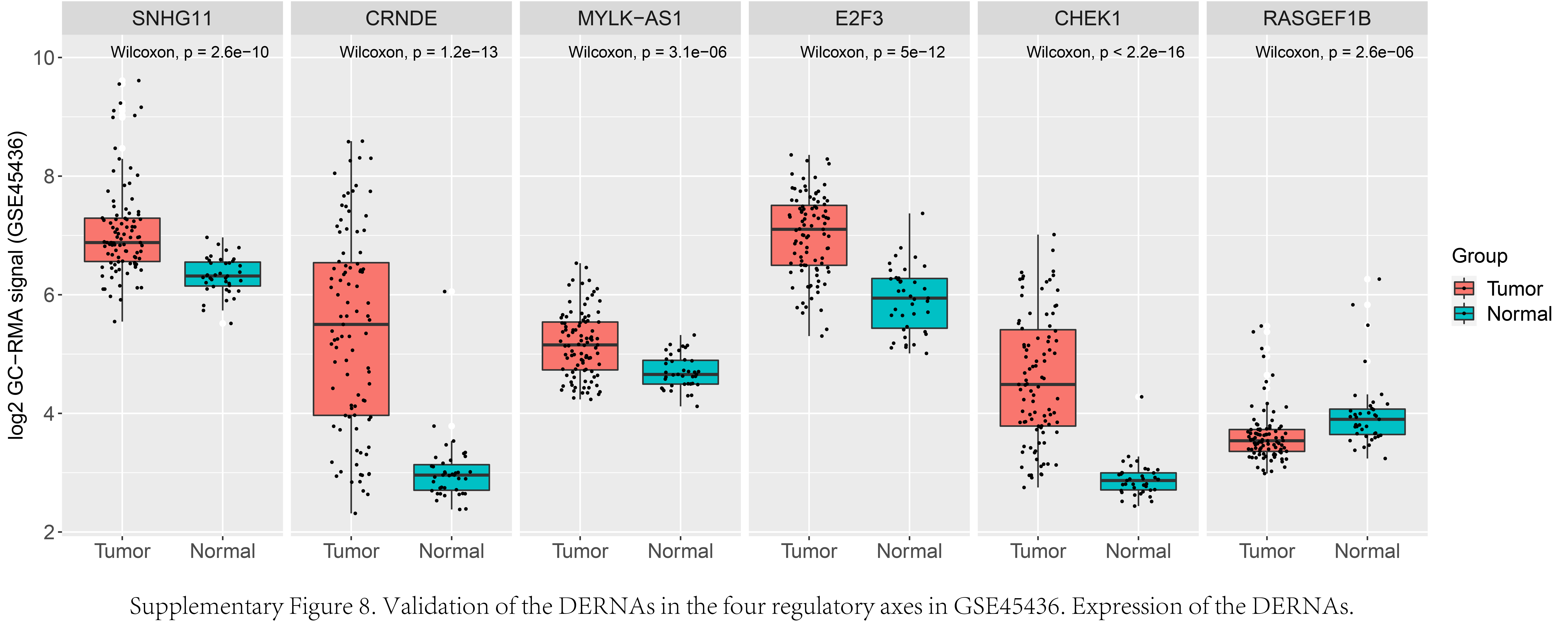

Supplement: Supplementary file 8 — Fig S8 [file CAM4-9-9219-s008.jpg]
